# Relationship between physical activity and the sense of coherence in healthy adults

**DOI:** 10.1016/j.aprim.2024.103106

**Published:** 2024-11-11

**Authors:** Miguel Jose Soares-Santeugini, Indira Enith Rodriguez-Prieto, Margareth Lorena Alfonso-Mora, Carolina Sandoval-Cuellar

**Affiliations:** a Universidad de La Sabana, Facultad de Enfermería y Rehabilitación, Grupo de Investigación Movimiento Corporal Humano, Chía, Colombia; b Universidad de Boyaca, Tunja Boyacá, Colombia

**Keywords:** Physiotherapy, Salutogenesis, Sense of coherence, Physical activity, Fisioterapia, Salutogénesis, Sentido de coherencia, Actividad física

## Abstract

**Objective:**

This study seeks to stablish a relation between the level of physical activity and the sense of coherence in young adults.

**Design:**

Cross sectional and analytical study in healthy young participants.

**Site:**

Web form application.

**Participants:**

191 active or inactive adult men or women between the ages of 18 and 45 without cognitive alterations. Professional or amateur athletes were excluded.

**Main measurements:**

Correlation between the sense of coherence instrument (SOC-13) and Inventory of Physical Activity Questionnaire (IPAQ) were applied.

**Results:**

The physical activity levels in the sample were distributed as: high level 34%, medium level 52%, and low level 13%. The mean of SOC-13 was 52.4. No difference was found when comparing between SOC-13 for each group of physical activity (high: 55, medium: 54, low: 58, *p* > 0.05), no correlation between SOC-13, age, and MET's reported by participants was found.

**Conclusions:**

In healthy individuals, the IPAQ measure of physical activity levels showed no correlation with the sense of coherence in healthy young adults. Apparently, a sedentary lifestyle does not correlate with an individual's self-directed pursuit of health. It is possible that being physically active or sedentary is related to extrinsic variables associated with culture or family environment.

## Abstract (es)

**Objetivo:**

Este estudio busca establecer una relación entre el nivel de actividad física y el sentido de coherencia en adultos jóvenes.

**Diseño:**

Estudio transversal y analítico en participantes jóvenes sanos.

**Emplazamiento:**

Aplicación de formulario web.

**Participantes:**

Ciento noventa y un hombres o mujeres adultos activos o inactivos de entre 18 y 45 años sin alteraciones cognitivas. Se excluyó a los deportistas profesionales o aficionados.

**Mediciones principales:**

Se aplicó la correlación entre el instrumento sentido de coherencia (SOC-13) y el cuestionario de Inventario de Actividad Física (IPAQ).

**Resultados:**

Los niveles de actividad física de la muestra se distribuyeron en: nivel alto 34% nivel medio 52% y nivel bajo 13%. La media del SOC-13 fue de 52,4. No se encontraron diferencias al comparar el SOC-13 para cada grupo de actividad física (alto: 55, medio: 54, bajo: 58 p > 0,05), no se encontró correlación entre el SOC-13, la edad y los MET reportados por los participantes.

**Conclusiones:**

En individuos sanos, la medida IPAQ de los niveles de actividad física no mostró correlación con el sentido de coherencia en adultos jóvenes sanos. Aparentemente, un estilo de vida sedentario no se correlaciona con la búsqueda autodirigida de la salud por parte del individuo. Es posible que ser físicamente activo o sedentario esté relacionado con variables extrínsecas asociadas a la cultura o al entorno familiar.

## Introduction

Doctor Aaron Antonovsky was the first person to describe the sense of coherence. The term Salutogenesis makes reference to the Pursuit of health of an individual on their own accord.^1^ This concept analyzes the potential that a person has of strengthening themselves personally and socially, thus protecting and promoting their own health.^2^ Among the conditions that modify Salutogenesis are Psychosocial, Physical and Biomechanical stressors, the former being the most studied. Physical and Biomechanical stressors are related to movement and Physical Activity (PA).^3^, ^4^, ^5^, ^6^, ^7^, ^8^

The salutogenic concept has been worked on in various professional fields. Different studies apply the concept in health such as Medicine, Nutrition, and Dentistry.^9^, ^10^, ^11^ It is used in that same way in order to improve the quality of life of people from the perspective of holistic medicine, trying to find alternative treatments vs. conventional ones.^12^ There have even been reviews of interventions that affect Salutogenesis.^13^ However, little is known about the influence that physical activity has on it.

The sense of coherence (SOC) has been identified as a protector of frailty in geriatric patients.^14^ Other works show that interventions based on the SOC model have a positive impact on self-efficacy, helping in the management and control of symptoms in patients with peritoneal dialysis.^15^ As well as Voseckova et al.^16^ having found that a high score of SOC is a useful tool for monitoring diabetic patients and their possible complications.

From mental health and psychology's point of view, there is evidence of a relationship between a low SOC and mental illnesses such as personality disorders,^17^ attention deficit and hyperactivity,^18^ and a low level of SOC. Similarly, high levels of stress can be associated with a lack of SOC.^19^ Even in dentistry, relationships have been established between SOC and esthetic self-perception of dental implants^9^ in adolescents closely related to a SOC. In addition, fear and anxiety associated with oral health in the pediatric population is related to a low SOC.

The importance of Salutogenesis and SOC in health, represents the capability to cope with life stressors and changes. Even in healthy people, adapting to different stressors or changes in life is crucial to maintaining and/or improving mental and physical health across the lifespan. As stated by Bauer et all., Salutogenesis is an important theoretical concept to support the future of primary care, this means that in a Heath System it should be mandatory to design interventions with the three global main concepts of the model: Comprehensibility, Manageability, and Meaningfulness in all aspects of life.^3^ The aim of this study is to identify the relationship between the level of physical activity and the sense of coherence in healthy individuals.

## Materials and methods

### Study design

This is a cross-sectional study; the information was collected through a free-access digital platform. The study was approved by the Research Ethics Committee (Faculty Nursing and Rehabilitation Universidad de La Sabana IRB # 011, August 2022). All participants were provided with online informed consent prior to enrollment in the study.

### Participants

The sample was intentionally selected for the researcher's convenience, representing a non-probabilistic approach, and comprised 200 healthy individuals. Out of that number, 9 patients were excluded due to incomplete required data in the survey. Therefore, the total population of the study was 191.^20^, ^21^ The inclusion criteria were active or inactive adult men or women between the ages of 18 and 45 without cognitive alterations that prevent them from filling out the forms. Professional or amateur athletes were excluded.

Access to the virtual survey was provided via an open-access link, which was distributed using a snowball sampling method. The collection process was carried out virtually through the Google Forms platform, in which the participants recorded the requested data regarding age, level of physical activity, and sense of coherence. The collecting of data took place over a 1-month period, in which the form was disseminated through social networks with convenience sampling.

### Measurement/data sources

The variables analyzed were physical activity (PA) and sense of coherence (SOC), alongside sociodemographic factors such as age and occupation. Each was assessed by means of a validated tool suitable for the population, being these IPAQ and SOC-13 respectively. The first one determines an average of the METs consumed over the last 7 days prior to answering the questionnaire. By determining the time spent on each of the activities (light, moderate and intense) and summing up the results, the individual is classified as having a Mild, Moderate, or Intense level of Physical Activity. The second consists of a series of 13 questions which measure the capacity to adapt to the various stressors that are found around a person. A higher score on the SOC scale suggests a stronger SOC, indicating that an individual is more likely to perceive their life as comprehensible, manageable, and meaningful. The score ranges from 13 to 91 points, and the treatment of this variable was quantitative.

SOC-13 aims to assess an individual's perceived ability to cope with and make sense of life's challenges. It focuses on understanding the factors that promote health and well-being rather than solely examining the causes of illness. The SOC analysis consists of three main components: *Comprehensibility* which refers to the extent to which individuals perceive their environment as understandable, structured, and predictable. It involves the capacity to make sense of the stimuli and events in one's life and to perceive them as coherent and meaningful. *Manageability*, relating to an individual's belief in their capacity to handle and manage stressful situations effectively. It encompasses the perception of available resources, both internal and external, that can be used to cope with challenges. *Meaningfulness*, life's meaning, purpose, and worth of engagement. It involves the ability to find significance and value in various aspects of life, such as work, relationships, and personal goals.^22^, ^23^, ^24^, ^25^, ^26^

The IPAQ questionnaire was designed to measure physical activity in four domains: work-related activity, transportation-related activity, household-related activity, and leisure-time related activity. It captures both moderate-intensity and vigorous-intensity physical activity, as well as walking. By assessing these different domains, the questionnaire aims to provide a comprehensive overview of an individual's overall physical activity patterns. The questionnaire typically consists of several sections, these are: *Demographic information*, *Physical activity assessment* (physical activity across the different domains. It involves asking participants to recall the duration and frequency of specific activities they engage in during a typical week), *Sedentary behavior assessment*, and finally the Scoring (providing an estimate of an individual's total physical activity level based on their responses). The scoring algorithm considers the intensity, frequency, and duration of reported activities to calculate a measurement of overall physical activity.^27^ The IPAQ data were processed quantitatively for METS, and ordinals for the classification of physical activity level (Mild, Moderate, or Intense level of Physical Activity).

### Statistical analysis

IBM SPSS 27.0 was used to analyze the data of study. All variables were described using frequencies, means, medians, and percentages. The Kolmogorov–Smirnov test was applied to assess the normality of the age, SOC-13 scores, and Total METs variables. Spearman's rank correlation coefficient was used to determine relationships between variables, while the Mann–Whitney *U* test was employed to compare differences in SOC scores across age groups. Finally, the IPAQ classification of physical activity levels was compared with SOC scores.

## Results

The characteristics of the total sample by reporting the age, occupation, physical activity level, total METs as well as the SOC-13 appear in Table 1.

**Table 1:** Descriptive analysis.

Characteristic	Total sample *n* = 191
*Age, median (SD)*	19 (6.397)

*Occupation*	
Student, *n* (%)	157 (82.20)
Health professionals, *n* (%)	10 (5.24)
Retailers and sales, *n* (%)	10 (5.24)
Clerk, *n* (%)	8 (4.19)
Engineer, *n* (%)	4 (2.09)
Sports, *n* (%)	2 (1.05)

*Physical activity level*	
High, *n* (%)	66 (34.6)
Moderate, *n* (%)	100 (52.4)
Low, *n* (%)	25 (13.1)

*Total METs, median (IQR)*	2024 (2161.39–2666.78)
*SOC-13 Result, mean (SD)*	55.42 (8.09)

The total METs reported in IPAQ questionnaire and calculating the sum of low, moderate, and intense physical activities. Median was calculated and reported 2024 METs (2161.39–2666.78) which results in a Moderated PA Performance of the full sample.^27^ PA was also calculated with the thresholds expressed being high (*n* = 66, 34.6%), moderate (*n* = 100, 52.4%) and low (*n* = 25, 13.1%). The SOC-13 mean was 55.42 (SD = 8.09).

Age and the IPAQ showed an inverse correlation (*R* −0.16, *p* 0.022), indicating that the older the age, the less physical activity, however, the strength of association is weak. Regarding SOC and METs, it had no association (*p* = 0.83). This suggests that the individual's perception ability to cope with and make sense of life's challenges does not depend of quantity of PA an individual practice weekly. Also, there is no relationship between age and SOC (*p* = 0.092) (Table 2).

**Table 2:** Correlational between the physical activity level – age and sense of coherence.

	Age	METs	SOC-13
SOC-13	0.09 (*p* 0.75)		
Age		***−0.16*** (p 0.022*)	
Mets			0.035 (*p* 0.831)

* Significance is *p* value <0.05.

The age variable was grouped into two segments 18–29 and the second group 30–45 years. The most notable group and the segment with the most participants relate to segment 18–29, the total number of participants was 173, this group reported a mean of SOC of 55 points in comparison to the 30–45 group with a mean of 57, there is no represent a difference between the age groups (*p* 0.229) (Table 3).

**Table 3:** SOC-13 by age ranges.

Age by ranges	*n* per age group	SOC-13 mean IC 95%	*p*
18–29, *n* (%)	173	55 (53.9–56.4)	0.229
30–45, *n* (%)	18	57 (53.81–61.19)	

Mann–Whitney *U* test significance 0.05.

**Figure 1:**
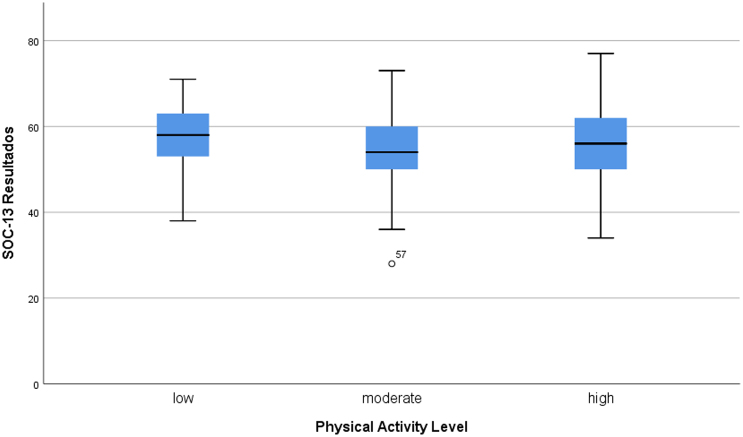
SOC-13 differences between SOC-13 and levels of physical activity.

The median comparison of SOC-13 between physical activity levels shown in Fig. 1 no difference in SOC-13 between low level (*n* = 29 SOC-13 = 58), median level (*n* = 104 SOC-13 = 54), and high level of PA (*n* = 65 SOC-13 = 56) was found.

## Discussion

This study was designed to establish a relation between the level of physical activity and the sense of coherence in young adults. The instruments used for this instance were SOC 13 and the IPAQ questionnaire. The main outcome of this study was not found correlation between sense of coherence and physical activity level.

These findings contradict the study by Adorni et al., which found that greater sense of coherence (SOC) was associated with higher levels of physical activity and improved quality of life in patients with cardiovascular disease. This discrepancy may be explained by the differences in the populations studied, as our research focuses on a different demographic group.^28^ Another study of Adorni et al. also shows that the SOC in the manageability dimension was associated with unhealthy diet and sedentary lifestyle. The same study reported that age does not adjust to the predictive models according to SOC, which is similar to our findings where no relationship was found between age and SOC. This topic in adolescents has shown association, those who are more physically active show higher scores in SOC.^29^ Along the same lines, the Riera-Sampol study also found an association between physical activity levels and SOC, with women having a greater risk of low physical activity who also showed lower SOC scores, which was also related to poor mental health.^30^ Engaging in physical activity can boost self-confidence and improve self-body image. Regular exercise leads to improvements in physical fitness, body composition, overall appearance, and mental and physical health.^31^, ^32^

The sense of coherence is a crucial factor that must be continually considered in the implementation of public health policies throughout a person's life. This involves acknowledging the social, environmental, and cultural contexts that shape individuals’ experiences. By promoting initiatives that support this holistic approach to development—particularly through encouraging physical activity—people can achieve a significantly better quality of life.^33^

Research consistently demonstrates that a stronger sense of coherence (SOC) is associated with better health outcomes across various populations, including older adults,^33^ adolescents,^29^ and women.^34^ Conversely, lower SOC levels correlate with poorer clinical conditions in areas such as eating disorders,^35^ anxiety,^30^ cardiovascular diseases,^28^ and cancer.^36^ Although the findings of this study do not definitively establish a relationship between the International Physical Activity Questionnaire (IPAQ) and SOC, existing literature suggests a potential link between a strong sense of coherence and the regular practice of physical activity.

One's occupation could also be a determining factor on how the sense of coherence. Each profession and work environment defer on their natures and requirements, such as responsibility, skills required and compensations, just to name a few. The way the person navigates adversity regarding their day-to-day activities is different in many ways. While some need the mental and physical capacities to execute live or death decisions, other face lesser conjectures. Nonetheless, concerning the occupation or job, making decisions is of crucial importance as it can have a significant impact on your overall well-being and prospects. SOC is susceptible to work-related changes and stresses, as well as the personal fulfillment and growth.^3^, ^37^, ^38^, ^39^, ^40^

Inactivity or a sedentary lifestyle has always been linked to various negative health outcomes. These prolonged periods of inactivity, related to work, health, cultural or social reasons, can lead to an increased risk of developing chronic conditions. As stated by Aaron Antonovsky, illness is one of the main reasons SOC is affected by a biological stressor. PA is key to reducing inactivity levels and therefore reducing the risk of incurring chronic illnesses that affect an individual's Salutogenesis. Thus, breaking the cycle of inactivity and adopting a physically active lifestyle can have a positive impact on both physical and psychological aspects of health.

However, we could not determine which of the main components of sense of coherence (Comprehensibility, Manageability, and Meaningfulness) is directly impacted by the physical activity level. Because of this, we recommend the following for future research, being the first: to develop a mixed method that allows a description of the importance of Physical Activity considering each of the main components of SOC. The second is to use the non-abbreviated version of SOC (SOC-29) to open the possibility of stating a relationship between the impact of the weekly Physical Activity and the main components of SOC. As stated by Antonovsky, there are Physical and Biomechanical stressors that may alter an individual's Salutogenesis.^3^

Some limitations of the study may be attributed to the data collection method. Conducting the survey through Google Forms could introduce accessibility bias and limitations inherent to self-administered surveys. For future research, we recommend that researchers consider a sample size of more than 300 participants. The results of this article include an analysis of the age variable, which shows a greater tendency toward the lower end of the spectrum. Therefore, we recommend that future research, in addition to grouping and analyzing data by age range, ensures similar data distributions across the age ranges included in the sample.

Salutogenesis is still a new concept that has only been explored in certain fields such as Psychiatry and Psychology, just to mention the most important. As stated by Antonovsky, the sense of coherence is affected by many factors in our day to day lives. Up until today, there are still many gaps in the knowledge about how those factors influence the main concepts of Salutogenesis.^3^, ^41^

## Conclusions

This study provides a comprehensive analysis of the relationship between physical activity (PA) and the sense of coherence (SOC) among adults, as detailed in the descriptive and relational analyses. The total METs reported in the IPAQ questionnaire, which included low, moderate, and intense physical activities, resulted in a median of 2024 METs (range: 2161.39–2666.78). This indicates a moderate PA performance across the sample. The distribution of PA levels revealed that 34.6% of participants engaged in high PA, 52.4% in moderate PA, and 13.1% in low PA.

The mean SOC-13 score was 55.42 (SD = 8.09). Only the main stressors (Psychosocial) have been more thoroughly studied, which leaves a lot of ground to be explored. Further examination of SOC-13 scores across different PA levels, illustrated in Fig. 1, revealed no significant differences between low (SOC-13 = 58), moderate (SOC-13 = 54), and high PA levels (SOC-13 = 56)

Analysis revealed a negative association between age and total METs (*R* = −0.16, *p* = 0.022), suggesting. The older the age, the less physical activity there is, with the strength of association being weak. However, no significant association was found between SOC and METs (*p* = 0.83), indicating that the perceived ability to cope with life's challenges does not depend on the PA quantity. Similarly, no significant relationship was found between age and SOC (*p* = 0.092).

In conclusion, our findings suggest that PA levels vary with age but do not significantly influence the sense of coherence. Similarly, SOC is not markedly affected by physical activity levels or age. These results contribute to our understanding of the interplay between physical activity and psychological resilience, highlighting areas for further research and intervention.

The study also highlights the need for further investigation into the mechanisms underlying the observed relationship. Future research should explore how various forms of physical activity influence different aspects of the sense of coherence and examine potential moderating factors, such as socio-cultural influences and individual differences.

**What is known about the topic?**

• The gap in knowledge on Salutogenesis lies in the lack of research focused on Physical Stressors. This leaves the job of developing the means to impact the well-being of patients based on Salutogenesis, to quite a few disciplines, such as Physical Therapy.
• The main findings demonstrate that no differences were found in the comparison between SOC-13 for each group of physical activity. This could change depending on many variables such as the age, sex, location, and work-related matters unique to each patient.
• Salutogenesis may improve the well-being of people and impact public health, by utilizing cost-effective strategies related to physical activity.

## Ethical considerations

Considering that the article involves the use of human subjects, the results presented in this article were carried out in accordance with the Code of Ethics of the World Medical Association (Declaration of Helsinki). The study was approved by the Research Ethics Committee (Faculty Nursing and Rehabilitation Universidad de La Sabana IRB # 011, August 2022). All participants were provided with online informed consent prior to enrollment in the study.

## Funding

This project did not receive any type of funding.

This project receives funding of Universidad de La Sabana ENF-66-2022 Call for the promotion of the teaching career and the categorization of researchers 2022.

## Authors’ contributions

MJS: conceptualization, methodology, formal analysis, investigation, resources, data curation, writing – original draft, writing – review & editing, visualization.

IER: conceptualization, methodology, writing – original draft, writing – review & editing, visualization, supervision.

MLA: conceptualization, methodology, writing – original draft, writing – review & editing, visualization, supervision.

CSC: methodology, review & editing, visualization, supervision.

## Conflict of interest

There was no conflict of interest among the authors.
